# Experts’ guidelines of intubation and extubation of the ICU patient of French Society of Anaesthesia and Intensive Care Medicine (SFAR) and French-speaking Intensive Care Society (SRLF): In collaboration with the pediatric Association of French-speaking Anaesthetists and Intensivists (ADARPEF), French-speaking Group of Intensive Care and Paediatric emergencies (GFRUP) and Intensive Care physiotherapy society (SKR)

**DOI:** 10.1186/s13613-019-0483-1

**Published:** 2019-01-22

**Authors:** Hervé Quintard, Erwan l’Her, Julien Pottecher, Frédéric Adnet, Jean-Michel Constantin, Audrey De Jong, Pierre Diemunsch, Rose Fesseau, Anne Freynet, Christophe Girault, Christophe Guitton, Yann Hamonic, Eric Maury, Armand Mekontso-Dessap, Fabrice Michel, Paul Nolent, Sébastien Perbet, Gwenael Prat, Antoine Roquilly, Karim Tazarourte, Nicolas Terzi, Arnaud W. Thille, Mikael Alves, Etienne Gayat, Laurence Donetti

**Affiliations:** 1 Service de réanimation médico-chirurgicale, hôpital Pasteur 2, CHU de Nice, 30, voie Romaine, 06000 Nice, France; 2 Unité CNRS 7275, Sophia-Antipolis, France; 3 Réanimation Médicale, centre hospitalier universitaire de Brest, La-Cavale-Blanche, 29609 Brest cedex, France; 4 Unitéde réanimation chirurgicale, service d’anesthésie-réanimation chirurgicale, pôle anesthésie-réanimations chirurgicales, Samu-Smur, hôpital de Hautepierre, hôpitaux universitaires de Strasbourg, 1, avenue Moliére, 67098 Strasbourg cedex, France; 5 Samu 93, hôpital Avicenne, 125, rue de Stalingrad, 93009 Bobigny, France; 6 EA 3509, UF recherche-enseignement-qualité, AP–HP, université Paris 13, Sorbonne Paris Cité, 125, rue de Stalingrad, 93009 Bobigny, France; 7 Department of medicine perioperative, centre hospitalier de Clermont-Ferrand, CHU Estaing, 1, rue Lucie Aubrac, 63100 Clermont-Ferrand, France; 8 Departement of anesthesiology and intensive care, hôpital Saint-Eloi, CHU de Montpellier, 80, avenue Augustin-Fliche, 34000 Montpellier, France; 9 Département d’anesthésie pédiatrique, hôpital d’enfants, 330, avenue de Grande-Bretagne TSA 70034, 31059 Toulouse cedex 9, France; 10 Kinésithérapie, réanimation Magellan, service d’anesthésie réanimation 2, hôpital Lévéque, avenue de Magellan, 33600 Pessac, France; 11 Department of Medical Intensive Care, Normandie University, Rouen University Hospital, 76000 Rouen, France; 12 UNIROUEN, EA-3830 Rouen, France; 13 Service de réanimation médicale et USC, CHU de Nantes, 30, boulevard Jean-Monnet, 44093 Nantes cedex, France; 14 Service d’anesthésie réanimation 3, anesthésie pédiatrique, hôpital des Enfants, CHU de Bordeaux, place Amélie-Raba-Léon, 33000 Bordeaux, France; 15 Service de réanimation médicale, hôpital Saint-Antoine, Assistance Publique–hôpitaux de Paris, 184, rue du Faubourg Saint-Antoine, université Pierre-et- Marie Curie, Paris, France; 16 UMR S 1136, Inserm et Sorbonne universités, UPMC université Paris 06, 75012 Paris, France; 17 Service de réanimation médicale, hôpitaux universitaires Henri-Mondor, AP–HP, DHU A-TVB, 94010 Créteil, France; 18 Université Paris Est-Créteil, faculté de médecine de Créteil, institut Mondor de recherche biomédicale, groupe de recherche clinique CARMAS, 94010 Créteil, France; 19 Service d’anesthésie réanimation pédiatrique, hôpital de la Timone, Assistance publique des Hôpitaux de Marseille, 264, rue Saint-Pierre, 13385 Marseille cedex, France; 20 Service de réanimation pédiatrique, hôpital des Enfants, CHU de Bordeaux, place Amélie-Raba-Léon, 33000 Bordeaux, France; 21 Réanimation médico-chirurgicale, CHU Gabril-Montpied, CHU Clermont-Ferrand, 63100 Clermont-Ferrand, France; 22 Réanimation médicale, Pôle ARSIBOU, CHU Cavale-Blanche, boulevard Tanguy Prigent, 29609 Brest cedex, France; 23 Anesthesiology and Intensive Care unit, CHU de Nantes, 44093 Nantes cedex, France; 24 Service des urgences-Samu, hôpital Edouard-Herriot, hospices Civils de Lyon, 69003 Lyon, France; 25 HESPER EA 7425, université Claude-Bernard Lyon 1, université de Lyon, 69008 Lyon, France; 26 Inserm, U1042, université Grenoble-Alpes, HP2, 38000 Grenoble, France; 27 Service de réanimation médicale, CHU de Grenoble Alpes, 38000 Grenoble, France; 28 Réanimation médicale, CHU de Poitiers, Poitiers, France; 29 Inserm CIC 1402 ALIVE, université de Poitiers, Poitiers, France; 30 Réanimation médico-chirurgicale, centre hospitalier intercommunal Poissy Saint-Germain-en-Laye, 10, rue du Camp-Gaillard, 78300 Poissy, France; 31 Department of anaesthesia an intensive care, hôpitaux universitaires Saint-Louis–Lariboisiére–Fernand-Widal, université Paris Diderot, Assistance Publique–Hôpitaux de Paris, Paris, France; 32 Unite’ 942 “Biomarker in CArdioNeuroVAScular diseases” Inserm, Paris, France; 33 Service USIR-SRPR, hospital de Forcilles, 77150, Férolles-Atilly, France

## Abstract

**Background:**

Intubation and extubation of ventilated patients are not risk-free procedures in the intensive care unit (ICU) and can be associated with morbidity and mortality. Intubation in the ICU is frequently required in emergency situations for patients with an unstable cardiovascular or respiratory system. Under these circumstances, it is a high-risk procedure with life-threatening complications (20–50%). Moreover, technical problems can also give rise to complications and several new techniques, such as videolaryngoscopy, have been developed recently. Another risk period is extubation, which fails in approximately 10% of cases and is associated with a poor prognosis. A better understanding of the cause of failure is essential to improve success procedure.

**Results and conclusion:**

In constructing these guidelines, the SFAR/SRLF experts have made use of new data on intubation and extubation in the ICU from the last decade to update existing procedures, incorporate more recent advances and propose algorithms.

## Introduction

Intubation and extubation of ventilated patients are not risk-free procedures on the intensive care unit (ICU) and can be associated with morbidity and mortality. Intubation in the ICU is frequently required in emergency situations for patients with an unstable cardiovascular system who may be hypoxic [1–3]. Under these circumstances it is a high-risk procedure with life-threatening complications (20–50%) such as hypotension and respiratory failure [2]. Technical problems can also give rise to complications. Generally three unsuccessful intubations [4] or two unsuccessful attempts at laryngoscopy are needed to justify the description *difficult airway*. These can make up 10–20% of intubations in the ICU and are associated with an increase in morbidity [2]. Several new techniques such as videolaryngoscopy have been developed for difficult airway management, but contrary to operating room practice, integrating these into ICU algorithms is not well established.

Another period of risk is extubation, which fails in approximately 10% and is associated with a poor prognosis [5, 6]. Extubation follows the successful weaning of patients from mechanical ventilation [7–9], but sometimes the re-establishment of spontaneous breathing is only possible with the tube in situ. An extubation failure is defined as the need for reintubation within 48 h of tube removal [7, 10], and the most recent consensus on weaning defined success as an absence of mechanical assistance for 48 h after extubation. There is a need to incorporate into these definitions the development of noninvasive ventilation (NIV) after extubation. NIV can be used as a weaning aid during extubation or as a preventive or curative treatment in acute respiratory failure occurring after extubation [11, 12]. As NIV can postpone the need for reintubation, a period of 7 days after extubation is required for a more accurate definition of failure [12]. To reduce the incidence of failure to extubate, the role of pathologies such as swelling and laryngeal edema in increasing risk must be appreciated. Screening for risk factors that might predispose to failure to extubate could improve the chances of success. In constructing these guidelines we have made use of new data on intubation and extubation in the ICU from the last decade to update existing procedures and incorporate more recent advances.

## Materials and methods

Table 1 represents a total of 19 experts were separated into 7 working groups (the pediatric experts being involved in all questions): The management of intubation has been assessed according to four headings: complicated intubation in the ICU, the materials required, pharmacology and the use of a management protocol. Extubation has been assessed according to three headings: prerequisites for extubation, extubation failure and the use of a management protocol. A specific analysis was performed for intubation and extubation in children.

**Table 1:** Guideline timeline

September 9, 2015	Start-up meeting
February 2016	Vote: first round
February 24, 2016	Postvote deliberation meeting
March 21, 2016	Vote: second round
June 2016	Amendment of two guidelines
September 2016	Guideline finalization meeting

As a first step, the organization committee defined the questions under consideration according to the PICO format (Patients Intervention Comparison Outcome). The system used to elaborate their recommendations is the GRADE^®^ method [13, 14].

These guidelines with their arguments were published in the journal Anaesthesia Critical Care and Pain Medicine [15, 16].

**Fig. 1:**
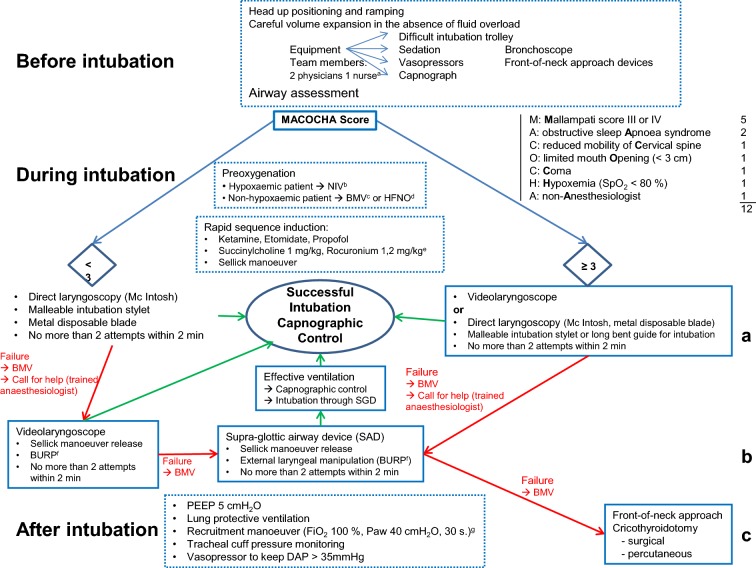
Algorithm for intubation

**Fig. 2:**
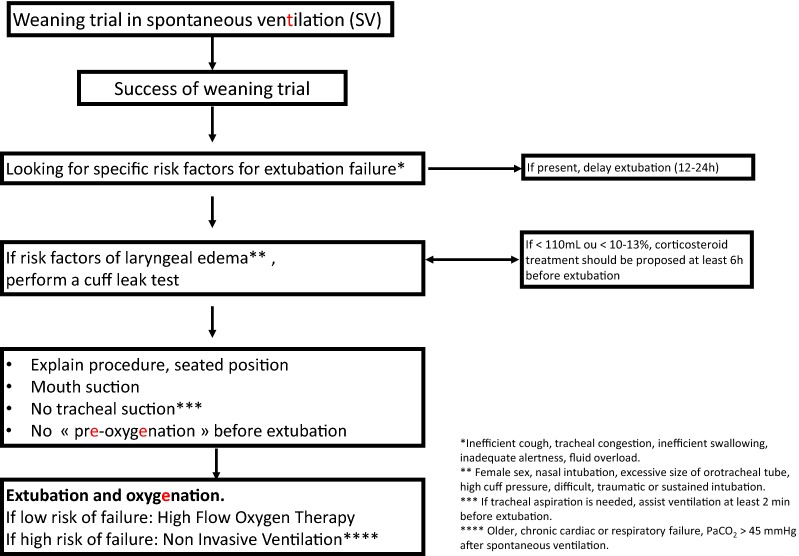
Algorithm for extubation

## Intubation of the ICU patient (Fig. 1)

### Complicated intubation in ICU

- R 1.1—All patients admitted to intensive care units must be considered at risk of complicated intubation. (Grade 1 +) Strong agreement.
- R 1.2—To reduce the incidence of complicated intubation, respiratory and haemodynamic complications must be anticipated and prevented, by careful preparing for intubation, and taking steps to maintain oxygenation and cardiovascular stability throughout the procedure. (Grade 1 +) Strong agreement.
- R 1.3—Risk factors of complicated intubation must be distinguished from predictive factors of difficult intubation. (Grade 1 +) Strong agreement.

### Intubation equipment

- R 2.1—Capnographic control of intubation in the intensive care environment is necessary to confirm the correct position of the endotracheal tube, the supraglottic device or the direct approach through the trachea. (Grade 1 +). Strong agreement.
- R 2.2—It is necessary to have a Difficult Airway Trolley and a Bronchoscope (conventional or single use) in intensive care units, for the immediate management of difficult intubation. (Grade 1 +) Strong agreement.
- R 2.3—Metal blades should be used for direct laryngoscopy in ICU to improve the success rate of endotracheal intubation. (Grade 1 +) Strong agreement.
- R 2.4—In order to limit intubation failures, videolaryngoscopes (VL) for intubation in intensive care must be used either initially or after failure of direct laryngoscopy. (Grade 2 +) Strong agreement.
- R 2.5—Supraglottic devices (SGD) must be used in the management of difficult intubation in intensive care, to oxygenate the patient, and facilitate intubation under bronchoscopic control. (Grade 1 +) Strong agreement.
- R 2.6—Theoretical and practical intubation knowledge must be acquired and diligently maintained (Grade 1 +) Strong agreement.

### Drugs and intubation of the ICU patient

- R 3.1—A hypnotic agent that facilitates rapid sequence induction (RSI) should probably be used (Etomidate, Ketamine, Propofol), the choice depending on medical history and the clinical situation of the patient. (Grade 2 +) Strong agreement.
- R 3.2—In critically ill patients, to facilitate tracheal intubation during RSI (rapid sequence induction), succinylcholine use is probably recommended (Grade 2 +) Strong agreement.
- R 3.3—Rocuronium at a dose above 0.9 mg/kg [1.0–1.2 mg/kg] should be used when succinylcholine is contraindicated. (Grade 1 +) Sugammadex should probably be rapidly available when rocuronium is used (Grade 2 +) Strong agreement.

### Protocols, algorithms and intubation of the ICU patient

- R 4.1—Non-invasive ventilation should probably be used for pre-oxygenation of hypoxaemic patients in ICU. (Grade 2 +) Strong agreement.
- R 4.2—It is possible to use high-flow nasal oxygen (HFNO) for pre-oxygenation in ICU, especially for patients not severely hypoxaemic. (Expert opinion) Strong agreement.
- R 4.3—A protocol for intubation including a respiratory component should probably be used in ICU to decrease respiratory complications. (Grade 2 +) Strong agreement.
- R 4.4—A post-intubation recruitment manoeuvre should probably be used in ICU in hypoxaemic patients, by integrating it into the respiratory component. (Grade 2 +) Strong agreement.
- R 4.5—A PEEP of at least 5 cmH2O should probably be applied after intubation of hypoxaemic patients. (Grade 2 +) Strong agreement.
- R 4.6—A cardiovascular component should probably be included in the protocol during intubation of ICU patients, by defining conditions of fluid challenge and early administration of amines to decrease cardiovascular complications. (Grade 2 +) Strong agreement.

## Extubation of the ICU patient (Fig. 2)

### Prerequisite

- R 5.1—We recommend a spontaneous breathing trial (SBT) before any extubation in an ICU patient ventilated for more than 48 h to decrease the risk of extubation failure. (Grade 1 +) Strong agreement.
- R 5.2—The SBT is inadequate as the sole means of detecting all patients at risk of extubation failure; before extubation we should probably screen for more specific causes and risk factors of failure including ineffective cough, excessive tracheo-bronchial secretions, swallowing disorders and altered consciousness. (Grade 2 +) Strong agreement.

### Extubation failure in ICU

- R 6.1—A cuff leak test should probably be performed before extubation to predict the occurrence of laryngeal oedema. (Grade 2 +) Strong agreement.
- R 6.2—A cuff leak test should be performed before extubation in ICU patients with at least one risk factor for inspiratory stridor to reduce extubation failure related to laryngeal oedema. (Grade 1 +) Strong agreement.
- R 6.3—Measures to prevent and treat laryngeal pathology should probably be implemented during mechanical ventilation. (Grade 2 +) Strong agreement.
- R 6.4—If the leak volume is low or nil, corticosteroids should probably be prescribed to prevent extubation failure related to laryngeal oedema. (Grade 2 +) Strong agreement.
- R 6.5—Once corticosteroid therapy is decided, it should be started at least 6 h before extubation to be effective. (Grade 1 +) Strong agreement.

### Respiratory therapy and extubation in the ICU

- R 7.1—As a prophylactic measure, we suggest high-flow oxygen therapy via a nasal cannula after cardiothoracic surgery. (Grade 2 +) Strong agreement.
- R 7.2—As a prophylactic measure, we suggest high-flow oxygen therapy via a nasal cannula after extubation in ICU for hypoxaemic patients and those at low risk of reintubation. (Grade 2 +) Strong agreement.
- R 7.3—As a prophylactic measure, we suggest the use of non-invasive ventilation after extubation in ICU for those at high-risk of reintubation, especially hypercapnic patients. (Grade 2 +) Strong agreement.
- R 7.4—As a therapeutic measure, we suggest the use of non-invasive ventilation to treat acute postoperative respiratory failure, especially after abdominal surgery or lung resection. (Grade 2 +) Strong agreement.
- R 7.5—As a therapeutic measure, we suggest that non-invasive ventilation not be used to treat acute respiratory failure after extubation in ICU, except in patients with underlying chronic obstructive pulmonary disease (COPD) or when there is obvious cardiogenic pulmonary oedema.(Grade 2-) Weak agreement.
- R 7.6—Treatment from a physiotherapist is probably required before and after endotracheal extubation following mechanical ventilation for more than 48 h to reduce the duration of weaning and the failure of extubation. (Grade 2 +) Strong agreement.
- R 7.7—A physiotherapist should probably attend endotracheal extubation, to limit immediate complications such as bronchial obstruction in patients with high risk of extubation failure. (Grade 2 +) Strong agreement.

## Pediatric specificity

### Intubation

#### Complicated intubation in pediatric intensive care unit (PICU)

- R 1.1 (pediatric)—All patients admitted in pediatric intensive care units must be considered at risk of complicated intubation. (Grade 1 +) Strong agreement.
- R 1.2 (pediatric)—To reduce the incidence of complicated intubation in pediatric intensive care unit, respiratory and hemodynamic complications must be anticipated and prevented, thanks to a carefully preparation of intubation, including preservation of oxygenation and hemodynamic throughout the procedure. (Grade 1 +) Strong agreement.
- R 1.3 (pediatric)—In pediatrics, risk factors of complicated intubation must be distinguished from predictive factors of difficult intubation. (Grade 1 +) Strong agreement.

### Intubation equipment

- R 2.1 (pediatric)—For child tracheal intubation in ICU, laryngoscopic blades suited to the habits of practitioners should be used (Miller straight blade or Macintosh curved blade). In case of exposition fail with the first blade, practitioner should change the type of blade for a new exposition. (Grade 2 +) Strong agreement.
- R 2.2 (pediatric)—In order to limit intubation failures in children, videolaryngoscopes (VL) for intubation in intensive care must be used either directly or after failure of direct laryngoscopy. (Grade 2 +) Strong agreement.
- R 2.3 (pediatric)—Oral intubation is probably preferred for children in intensive care units (Grade 2 +) Strong agreement.
- R 2.4 (pediatric)—cuffed tubes are likely to be used for children in intensive care units in order to limit the number of reintubations for leakage (Grade 2 +) Strong agreement.

### Drugs and intubation of the ICU patient

- R 3.1 (pediatric)—Hypnotic agent should probably be chosen allowing rapid sequence induction (Etomidate, Ketamine, Propofol) depending on medical history and clinical situation of the patient in pediatric ICU. Grade 2 + Strong agreement.
- R 3.3 (pediatric)—Succinylcholine is probably the first-line agent of choice for RSI in pediatric ICU patients with vital signs of distress. Rocuronium at a dose above 0.9 mg/kg [1.0–1.2 mg/kg] should be used when succinylcholine is contraindicated. (Grade 1 +) Sugammadex should probably be rapidly available when rocuronium is used. (Grade 2 +) Strong agreement.

### Bundles and intubation in PICU

- R 4.1 (pediatric)—Atropine should probably be administered before intubation during induction in PICU for children aged of more than 28 days to 8 years. Especially in children with septic shock, hypovolemia or when suxamethonium is used. (Grade 2 +) Strong agreement.

### Extubation

#### Prerequisite

- R 5.1 (pediatric)—A spontaneous breathing trial (SBT) should probably be performed before any extubation in PICU ventilated patient to decrease the risk of extubation failure. (Grade 2 +) Strong agreement.
- R 5.2 (pediatric)—The SBT being not sufficient by itself to detect all patients at risk for extubation failure, more specific causes and risk factors for extubation failure including ineffective cough, excessive tracheo-bronchial secretions, swallowing disorders, altered consciousness and some pediatrics specific factors should probably be screened before extubation. (Grade 2 +) Strong agreement.

### Extubation failure in ICU

- R 6.5 (pediatric)—When a corticosteroid therapy is decided, it should be started at least 24 h before extubation to be effective. (Grade 1 +) Strong agreement.

### Bundles of extubation in PICU

- R7 (pediatric)—We should probably not use NIV after extubation in pediatric ICU in low risk patients. (Expert advice) Strong agreement.
